# The role of conserved proteins DrpA and DrpB in nitrate respiration of *Thermus thermophilus*

**DOI:** 10.1111/1462-2920.14400

**Published:** 2018-10-02

**Authors:** Zahra Chahlafi, Laura Alvarez, Felipe Cava, José Berenguer

**Affiliations:** ^1^ Centro de Biología Molecular Severo Ochoa Universidad Autónoma de Madrid‐Consejo Superior de Investigaciones Científicas Madrid 28049 Spain; ^2^ Department of Molecular Biology Umeå University Umeå 901 87 Sweden

## Abstract

In many *Thermus thermophilus* strains, nitrate respiration is encoded in mobile genetic regions, along with regulatory circuits that modulate its expression based on anoxia and nitrate presence. The oxygen‐responsive system has been identified as the product of the *dnrST* (*dnr*) operon located immediately upstream of the *nar* operon (*narCGHJIKT*), which encodes the nitrate reductase (NR) and nitrate/nitrite transporters. In contrast, the nature of the nitrate sensory system is not known. Here, we analyse the putative nitrate‐sensing role of the bicistronic *drp* operon (*drpAB*) present downstream of the *nar* operon in most denitrifying *Thermus* spp. Expression of *drp* was found to depend on the master regulator DnrT, whereas the absence of DrpA or DrpB increased the expression of both DnrS and DnrT and, concomitantly, of the NR. Absence of both proteins made expression from the *dnr* and *nar* operons independent of nitrate. Polyclonal antisera allowed us to identify DrpA as a periplasmic protein and DrpB as a membrane protein, with capacity to bind to the cytoplasmic membrane. Here, we propose a role for DrpA/DrpB as nitrate sensors during denitrification.

## Introduction

The biosphere's nitrogen budget is maintained by denitrification performed by bacteria and archaea (Houlton and Bai, 2009; Ollivier *et al*., 2011; Martınez‐Espinosa *et al*., 2011; Fowler *et al*., 2013). Denitrification is a complex anaerobic respiration pathway driven through four enzymatic steps, in which nitrogen oxides are successively reduced from nitrate to nitrite, nitric oxide, nitrous oxide and finally dinitrogen by consecutive action of the corresponding Nar, Nir, Nor and Nos reductases. As oxygen is a better electron acceptor, expression of the denitrification enzymes is subjected to hierarchical transcriptional control by oxygen, with the presence of an appropriate nitrogen oxide also required (Zumft, 1997; Richardson and Watmough, 1999; Gaimster *et al*., 2018).

In *Escherichia coli*, expression of the main respiratory nitrate reductase (NR) depends on activation of the FNR protein via incorporation of a 4Fe‐4S iron–sulfur center through the iron–sulfur cluster (Isc) assembly proteins (Crack *et al*., 2008; Mettert and Kiley, 2015). FNR is a transcription regulator of the c‐AMP‐binding protein family CRP, which upon anaerobic activation dimerizes and binds to the promoters involved in anaerobic metabolism, such as that of the NR (Scott *et al*., 2003). However, this binding is not sufficient and further binding of the phosphorylated form of the response regulator NarL to upstream regions of the promoter is also required for effective recruitment of the RNA polymerase and further transcription of the NR operon. NarL is phosphorylated by dimers of its membrane‐bound kinase partner NarX when nitrate is present in the periplasm (Stewart, 2003). Homologues to the NarX/NarL two‐component system are present in most nitrate‐respiring species, constituting the most frequent nitrate sensory system for denitrification. However, other denitrifying bacteria exist which lack NarX/NarL homologues, suggesting that yet‐unknown alternative mechanisms for nitrate sensing must exist.

*Thermus thermophilus* is one of the cases in which no nitrate‐sensing homologues have been identified, though some strains of this phylogenetically ancient bacterium can use nitrogen oxides as electron acceptors in anaerobic conditions (Cava *et al*., 2008). In *T. thermophilus* NAR1, nitrate is reduced only to nitrite allowing its anaerobic growth (Ramírez‐Arcos *et al*., 1998b). This capability is encoded by a mobile genetic element (NCE, Nitrate Respiration Conjugative Element) (Cava *et al*., 2007), which includes at least three operons. The *nar* operon (*narCGHIJKT*) encodes the NarCGHI hetero‐tetrameric NR, a chaperone (NarJ), and the NarK and NarT nitrate/nitrite transporters. A second operon (*nrcDEFN*) codes for a dedicated hetero‐tetrameric NADH dehydrogenase (Cava *et al*., 2004b), while a third (*dnrST*) immediately upstream of *nar* codes for master regulatory genes that modulate expression of the NCE during anoxia and presence of nitrate. From the master regulators, DnrT is a cytoplasmic CRP‐like transcription factor required for induction of the promoters of the *nar* (*Pnar*), *nrc* (*Pnrc*) and *dnr* (*Pdnr*) operons on a concentration‐dependent basis, with activity *in vitro* independent of the presence of oxygen and/or nitrate (Cava *et al*., 2007). DnrS is also a cytoplasmic transcription factor required for expression from *Pnar* and its own *Pdnr* promoter, but it is not required for activation of the *Pnrc* promoter. Considering that no FNR‐like sensor is encoded by *T. thermophilus* and that DnrS is sensitive to oxygen, likely through a redox centre located at its N‐terminal GAF domain, we hypothesized that its function was to prevent activation of nitrate respiration under aerobic conditions (Cava *et al*., 2007). This unusual mechanism reflects a clear difference in the nitrate respiration regulatory circuits of *T. thermophilus* compared to those of well‐known models such as *E. coli* (and other Enterobacteria), which are mainly constituted by FNR‐like sensors (Crack *et al*., 2008).

Given these differences, it is not surprising that the mechanisms involved in nitrate sensing must also be different in *T. thermophilus*. In fact, NarX/NarL two‐component system is absent from all the *T. thermophilus* denitrifying strains so far published. However, our previous experiments have demonstrated that a sensory system for nitrate is transferred horizontally to the aerobic strain HB27 along with the NCE, supporting the existence of a sensory system unrelated to NarX/NarL that is genetically linked to the *nar* operon (Ramírez‐Arcos *et al*., 1998b; Alvarez *et al*., 2014b). Analysis of the sequences from different denitrifying strains of *Thermus* identified a two‐gene operon (*drpAB*) located immediately downstream of the *nar* operon in all nitrate‐reducing strains of *Thermus*. In this article, we have analysed the role of these two genes in the nitrate‐respiring model strain *T. thermophilus* NAR1. Our data suggest that these genes are involved in the expression of the NR operon, likely through the periplasmic detection of nitrate by DrpA.

## Results

### *Conserved genes are encoded downstream the respiratory NR*

**Figure 1:**
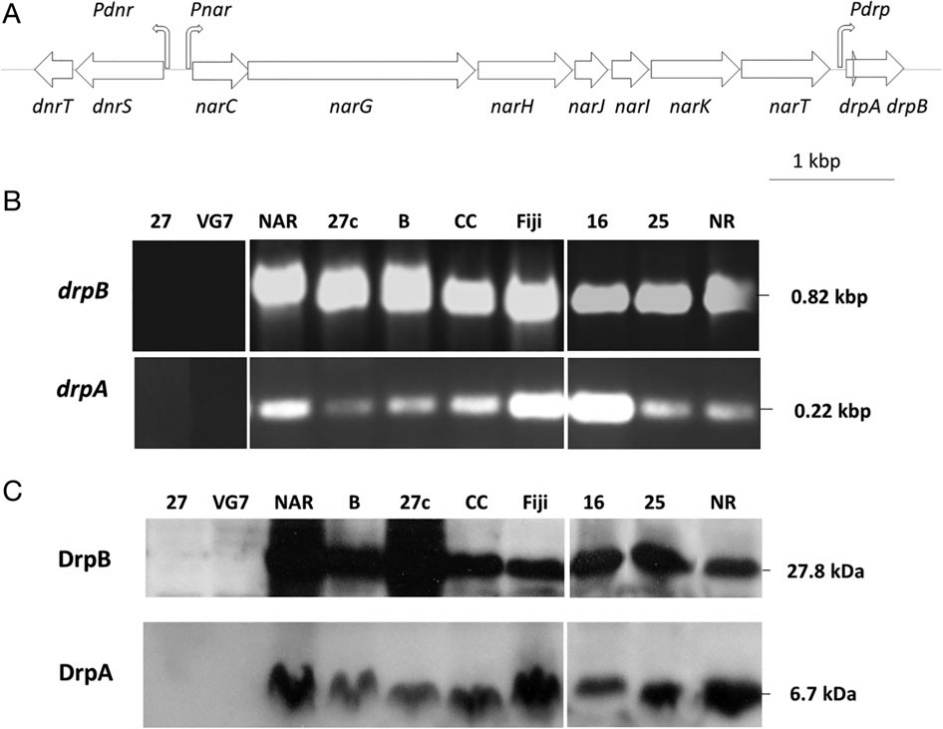
Expression of DrpA and DrpB in denitrifying strains of *T. thermophilus*.A. Scheme of the *nar*, *dnr* and *drp* operons.B. PCR assays on total DNA of the T. thermophilus strains HB27 (27), VG7 (VG7), NAR1 (NAR), HB27c (27c), B (B), CC16 (CC), Fiji3A1 (Fiji), PRQ16 (16), PRQ25 (25) and NR17 (NR). Primers were the same used to clone the *drpA* and *drpB* genes (Supporting Information Table S2), and the lanes with the PCR products correspond to two (*drpB*) and one (*drp*A) agarose gels with lanes ordered to fit image in panel (C).C. Western blot to detect DrpA and DrpB in the same strains of T. thermophilus, incubated for 12 h under anaerobic conditions with 20 mM nitrate.

The operon for the respiratory NR of different *Thermus* spp. ends with a gene encoding a transporter of the MFS family (Alvarez *et al*., 2014b) followed by a putative Rho‐independent transcription terminator (Fig. 1A). Immediately downstream of its coding sequence, there are overlapping genes that encode two highly conserved proteins with small sizes (Supporting Information Fig. S1) (Alvarez *et al*., 2014b). In *T. thermophilus* NAR1, the coding sequence of the first conserved protein (named Denitrification regulatory protein A, DrpA) includes three putative ATG start codons located 51, 89 and 128 bp downstream of NarT stop codon, the last gene of the *nar* operon. The different starting sites could produce alternative proteins of 101 (11,297 Da), 85 (9,589 Da) and 72 (8,276 Da) amino acids. A second putative protein of 273 (30,632 Da) amino acids (DrpB) is encoded by a gene overlapping *drpA* by 25 bp.

A search in GenBank revealed the existence of DrpA and DrpB homologues in nitrate respiration clusters of different *Thermus* spp. and other phylogenetically related genera such as *Meiothermus silvanus*, as previously noted (Alvarez *et al*., 2014b). Based on sequence alignments and conservation of DrpA (Supporting Information Fig. S2), the third putative start codon seems to be the most likely origin of translation. DrpA homologues exhibit a conserved domain with unknown function (DUF2249) that extends throughout most of the protein sequence. This domain was present in approximately 3,500 proteins either as a single domain (2,500 proteins) or duplicated (300 proteins). The DUF2249 domain was also found in combination with hemerytrin domains, involved in oxygen transport in invertebrates and likely in prokaryotes (French *et al*., 2008), and also with domains present in response regulators of bacterial two‐component systems that control secondary metabolism and virulence in different bacteria. Similarly, DrpB had homologues also in several *Thermus* spp. and related genera (Supporting Information Fig. S3). The protein is composed of three conserved domains of unknown function: an N‐terminal DUF1858 domain and two tandem DUF2249 domains predicted in the middle part and at the C‐terminus of the protein. The combination of these three domains appeared in 93 proteins in the InterPro repository (Finn *et al*., 2017), all without known function.

### *The drp genes are expressed in nitrate respiring T. thermophilus spp*.

In order to determine if the *drp* genes were conserved in yet‐unsequenced strains of *T. thermophilus*, PCR assays were carried out on genomic DNA from nitrate‐respiring and denitrifying strains VG7, B, CC16, Fiji3A, PRQ16, PRQ25 and NR17 using specific primers for the amplification of *drpA* and *drpB* (Supporting Information Table S2). Both genes were detected in all these strains except for VG7 (Fig. 1B). It is important to note that in VG7, NR activity was detected but not by PCR (Cava *et al*., 2008).

To further confirm expression of the detected *drp* genes, polyclonal rabbit antisera were first produced against purified recombinant N‐terminal His‐tagged fusions of DrpA and DrpB proteins from *T. thermophilus* NAR1 (Supporting Information Fig. S4). Western blots were performed on total cell extracts from the above strains cultured under anoxic conditions with nitrate for 4 h. Proteins of expected sizes (6.7 and 30 kDa for DrpA and DrpB, respectively) were detected in all strains in which the corresponding genes were identified by PCR (Fig. 1C). It is important to note that the proteins were not detected in the aerobic strain HB27 (lane 27), whereas they were identified in its nitrate‐respiring derivative HB27c (lane 27c), which was obtained by conjugation with the NAR1 strain (Ramírez‐Arcos *et al*., 1998b), demonstrating that the *drp* genes were co‐transferred along with the *nar* operon. In conclusion, DrpA and DrpB were shown to be actual proteins expressed in most nitrate‐respiring strains of *T. thermophilus*.

### *drpAB are expressed as a bicistronic messenger from its own promoter under nitrate respiration*

**Figure 2:**
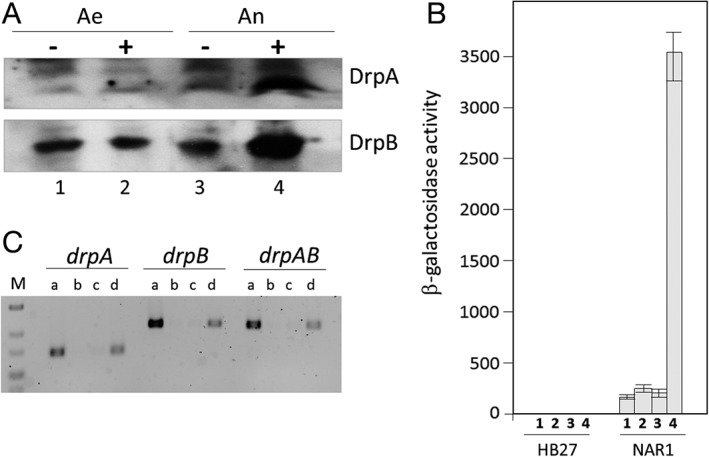
Expression of DrpA and DrpB in nitrate respiration conditions.A. Western blot to identify DrpA and DrpB in cultures of T. thermophilus NAR1 grown aerobically (Ae) or incubated 4 h anaerobically (An) in the presence (+) or absence (−) of 20 mM nitrate.B. Beta galactosidase activity produced by plasmid pMHPdrpBgal in T. thermophilus NAR1 under the following conditions: aerobiosis without (1) or with (2) 20 mM nitrate, and after 4 h of anaerobic growth without (3) or with (4) 20 mM nitrate. The data are mean values of nine samples from three independent experiments.C. RT‐PCR of RNA isolated from cultures of T. thermophilus NAR1 grown anaerobically with 20 mM nitrate for 4 h: a) control PCR on DNA; b) PCR on RNA without the reaction mix for reverse transcription (RT); c) PCR on RNA with the reaction mix for RT but without incubation for the RT step; and d) RT‐PCR on RNA. The primers used to amplify *drpA*, *drpB* and an intergenic region (*drpAB*) are indicated in Supporting Information Table S2.

To check if the expression of DrpA and DrpB required the same environmental conditions as those required for the expression of the *nar* operon, the presence of both proteins was followed by western blot in *T. thermophilus* NAR1 cultures grown under different conditions. During aerobic growth, only a small amount of DrpB and even less DrpA was detected, independent of the presence or absence of nitrate (Fig. 2A, lanes 1 and 2). In contrast, anaerobic conditions did not induce the expression of protein, unless nitrate was also present (Fig. 2A, lanes 3 and 4).

To determine if a specific promoter (*Pdrp*) was responsible for this induction, the region immediately upstream of *drpA* was cloned into a promoter probe vector and assayed for its expression under the same conditions. The *Pdrp* promoter was not active in any condition in the aerobic strain HB27, whereas in the NAR1 strain, the promoter was induced at very low levels with oxygen (Fig. 2B, lanes 1 and 3) or even under anaerobic conditions without nitrate (Fig. 2B, lane 2). Under anoxia plus nitrate conditions, the *Pdrp* promoter was strongly activated (Fig. 2B, lane 4).

To further confirm that both genes were being co‐transcribed, RNA was purified from an induced culture and tested by RT‐PCR assays with appropriate primers, revealing positive mRNA signals for each individual gene and for a region spanning both genes (Fig. 2C). Therefore, both *drp* genes are expressed from a dedicated promoter as a bicistronic mRNA with the same conditions as required for the expression of the NR.

### *Drp proteins are required for efficient nitrate respiration*

To further analyse the role of Drp proteins, single and double‐knockout deletion mutants of the *drpA* and *drp*B genes were obtained in a NAR1 genetic background; the scheme used to construct the mutants is shown in Supporting Information Fig. S5.

**Figure 3:**
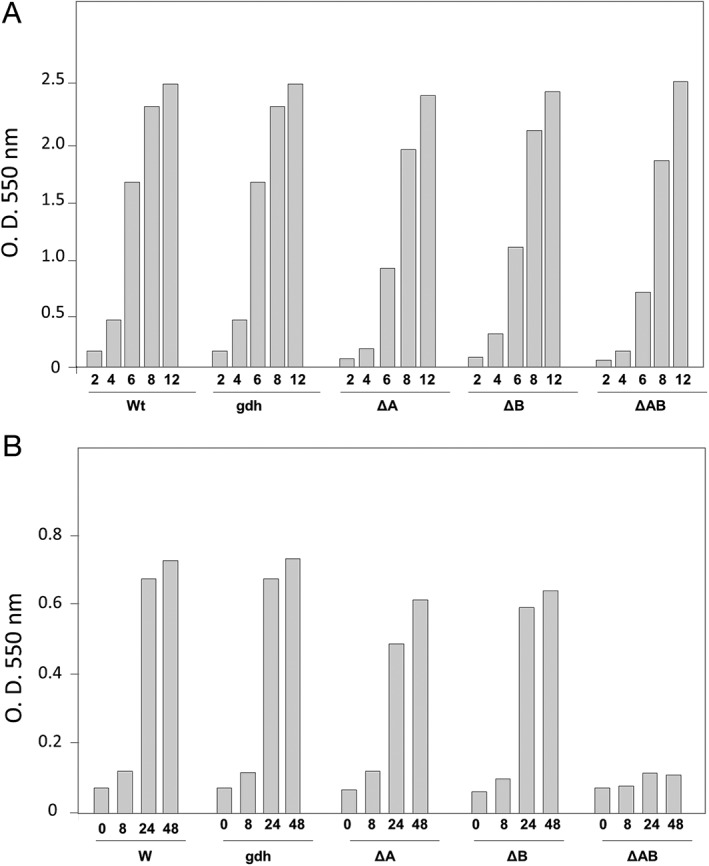
Growth of *drp* mutants.A. Aerobic growth of the NAR1 (wt) strain, its derivatives *gdh::kat* (gdh), *drpA* (ΔA), *drpB* (ΔB) and *drpAB* double mutant (ΔAB).B. Anaerobic growth of the same strains with 20 mM nitrate. Bars correspond to OD_550_ reached at the indicated time (h).

When the growth of the selected NAR1 mutants was tested, all of them were capable of growth in aerobic conditions similar to the wt NAR1 or a *gdh::kat* derivative used as a Km‐resistant wt control (Fig. 3A). Under anaerobic conditions with nitrate, Δ*drpA* or Δ*drpB* single mutants were able to grow at similar rates to the control strains (Fig. 3B), whereas the Δ*drpAB* double mutant grew to a much lower density after 48 h. Therefore, deletion of any single gene had a mild effect on anaerobic growth, whereas the absence of both proteins resulted in a loss of anaerobic growth with nitrate.

### *Effect of drp mutants on expression of the NR*

**Figure 4:**
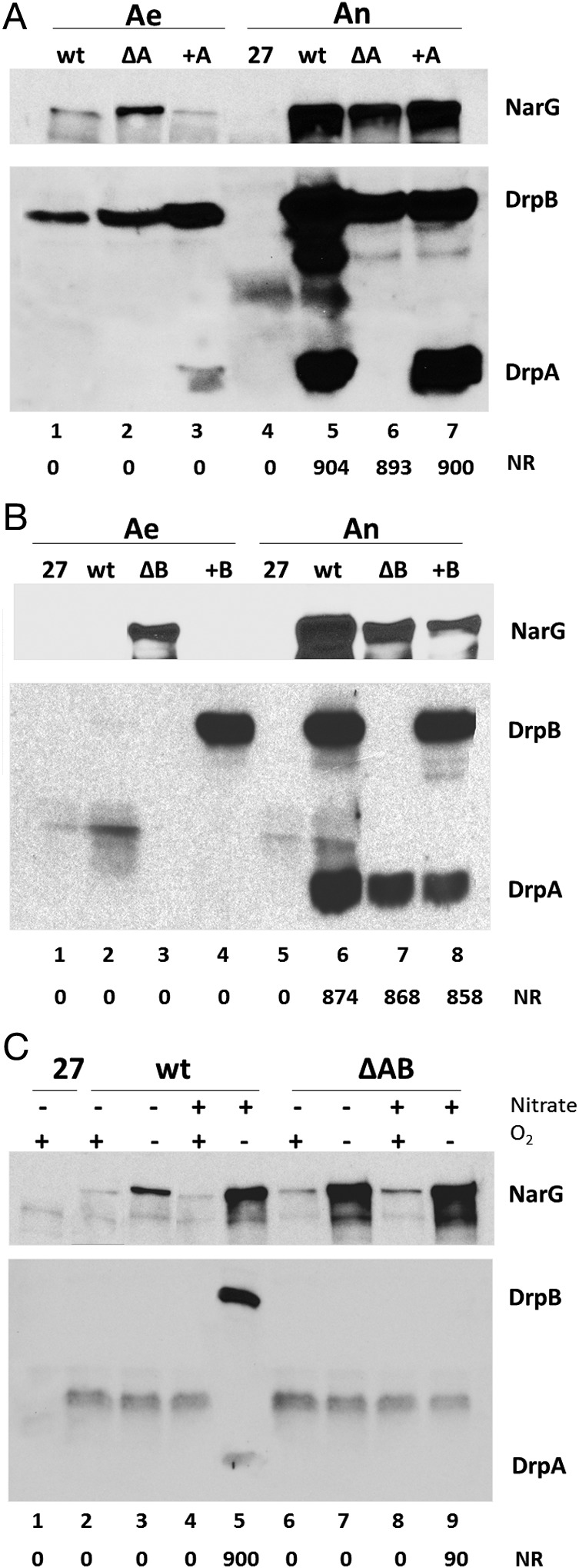
Effect of the absence of DrpA or DrpB on expression of the nitrate reductase. A. Cultures of the wild‐type NAR1 *gdh::kat* strain (wt) and the *drpA* (ΔA) mutant transformed with an empty pWUR plasmid, and the *drpA* mutant transformed with a pWUR derivative that overexpresses DrpA (+A) were grown under aerobic conditions (Ae) or incubated for 12 h under anaerobic conditions with 20 mM nitrate (An). NarG, DrpA and DrpB were detected by western blot. B. Cultures of the wild‐type NAR1 *gdh::kat* strain (wt) and the *drpB* (ΔB) mutant transformed with an empty pWUR plasmid and the *drpB* mutant transformed with a pWUR derivative that overexpresses DrpB (+B) were grown under aerobic conditions (Ae) or incubated for 12 h under anaerobic conditions with 20 mM nitrate (An). NarG, DrpA and DrpB were detected by western blot. C. Effect of the Drp proteins on expression of the nitrate reductase. Cultures of the wild‐type NAR1 *gdh::kat* strain (wt) and the *ΔdrpAB* (ΔAB) mutant were grown aerobically or anaerobically in the presence (+) or absence (−) of 20 mM nitrate. NarG, DrpA and DrpB were detected by western blot. Nitrate reductase (NR) activity is expressed as enzymatic units under each lane.

The putative effect of DrpA and DrpB on expression of the major subunit of the NR (NarG) was assayed by western blot with specific rabbit antisera (Fig. 4). As the maturation of this enzyme is a complex process that involves cofactors and chaperons that could be also affected by the absence of any of these enzymes, the NR activity was also assayed. In parallel, the presence and relative amounts of the Drp proteins were also tested by western blot with a mix of anti‐DrpA and anti‐DrpB antisera, in which each protein could be specifically identified by its size in the same assay. An unrelated protein of intermediate size was also detected by these antisera in the wild type and in the double drpAB mutant and, even in the aerobic HB27 strain, but without affecting the detection of DrpA and DrpB. As shown in Fig. 4, the three proteins NarG, DrpA and DrpB were induced under anoxia plus nitrate compared to their respective controls grown under aerobic conditions without nitrate, although the expression level detected for each protein varies under the latter condition.

The effects of the absence of DrpA are shown in Fig. 4A. DrpA was not detected under aerobic conditions in the wild‐type strain (lane 1) but was detected in cultures grown under anoxia plus nitrate (lane 5). As expected, DrpA was not detected in the Δ*drpA* mutant (lanes 2 and 6) except for when it was supplied *in trans* (lanes 3 and 7). Under aerobic conditions without nitrate, a small amount of NarG was detected in the *drpA* mutant (lanes 1 vs. 2), but without NR activity. Under anoxia with nitrate NarG was overexpressed in an apparently lesser amount in the *drpA* mutant compared to the wild type (lanes 6 vs. 5), although the NR activity was similar. Expression of DrpA *in trans* produced similar NarG induction as in the wild‐type strain (lane 7). Thus, DrpA absence has apparently moderate effects on NarG expression.

The effects of DrpA absence on DrpB expression were moderate also. The amount of DrpB detected under aerobic conditions was higher than in the wild type (lane 2), but in this case, a putative polar effect produced by the insertion of the *kat* gene cannot be disregarded. Despite this, DrpB was induced under anoxic conditions with nitrate (Fig. 4A, lane 6), but also at apparently slightly lower levels than in the wild type (Fig. 4A, lane 5). DrpB levels increased when DrpA was supplied in trans both under aerobic conditions (lane 3) and under anoxia plus nitrate (lane 7). Noteworthily, the amount of DrpA detected in aerobic cultures when over‐expressed from a plasmid was much lower than expected and lower than the amount detected under inducing conditions in the wild type, suggesting that DrpA may be degraded under aerobic conditions (compare lanes 3 and 7), similarly to master regulator DnrS (Cava *et al*., 2007).

In contrast to the mild effect of the absence of DrpA, a more dramatic effect on NarG expression was observed when DrpB was not present (Fig. 4B). In Δ*drpB* mutants, high levels of NarG were detected under aerobic conditions, but without detectable NR activity (lane 3), whereas NarG was not detected under these same conditions when DrpB was constitutively expressed from a plasmid (lane 4). Under anaerobic conditions with nitrate, expression of NarG in the Δ*drpB* mutant was lesser than in the wild type (compare lanes 6 and 7) and overexpression of DrpB from a plasmid even decreased the amount of NarG detected under these same conditions. It is interesting to note that as with the Δ*drpA* mutant, the NarG expressed under aerobic conditions in the Δ*drpB* mutant was not active at all.

When the Δ*drpAB* mutant was assayed (Fig. 4C), expression of NarG was clearly depending on oxygen absence (compare lanes 6 vs. 7 and 8 vs. 9) but became basically independent of nitrate, in such a way that cells incubated under anoxia without nitrate overexpressed the protein apparently in similar amounts than those in anoxia with nitrate (compare lanes 7 and 9), whereas in the wild‐type strain, the induction of NarG under anoxia by the presence of nitrate was evident (compare lanes 3 and 5). Interestingly, in the double mutant, the NR activity expressed without nitrate had no activity at all and that expressed with nitrate showed basically one‐tenth (90 ± 26 U) of that reached by the wild type (900 ± 52 U), despite the fact that there was apparently less NarG in the latter, thus explaining the low efficiency of the Δ*drpAB* mutant to grow anaerobically (Fig. 3B).

In conclusion, these data implicate DrpA and DrpB in the expression of the NR, suggesting that in the absence of both proteins, NarG expression becomes independent of the presence of nitrate.

### *Effects of Drp proteins on nitrate respiration promoters*

The above results suggest a role for DrpA and DrpB in the expression of the NR. In order to determine if the effect is exerted at the transcriptional level, promoter probe plasmids were employed under four culture conditions: aerobic (1), anaerobic (2), aerobic with nitrate (3) and anaerobic with nitrate. In addition to the promoter of the NR (*Pnar*), the *Pdnr* promoter was included in these assays, which controls expression of the master regulators DnrS and DnrT, to determine if the effects detected regarding NarG expression could be an indirect consequence of effects on these regulators.

**Figure 5:**
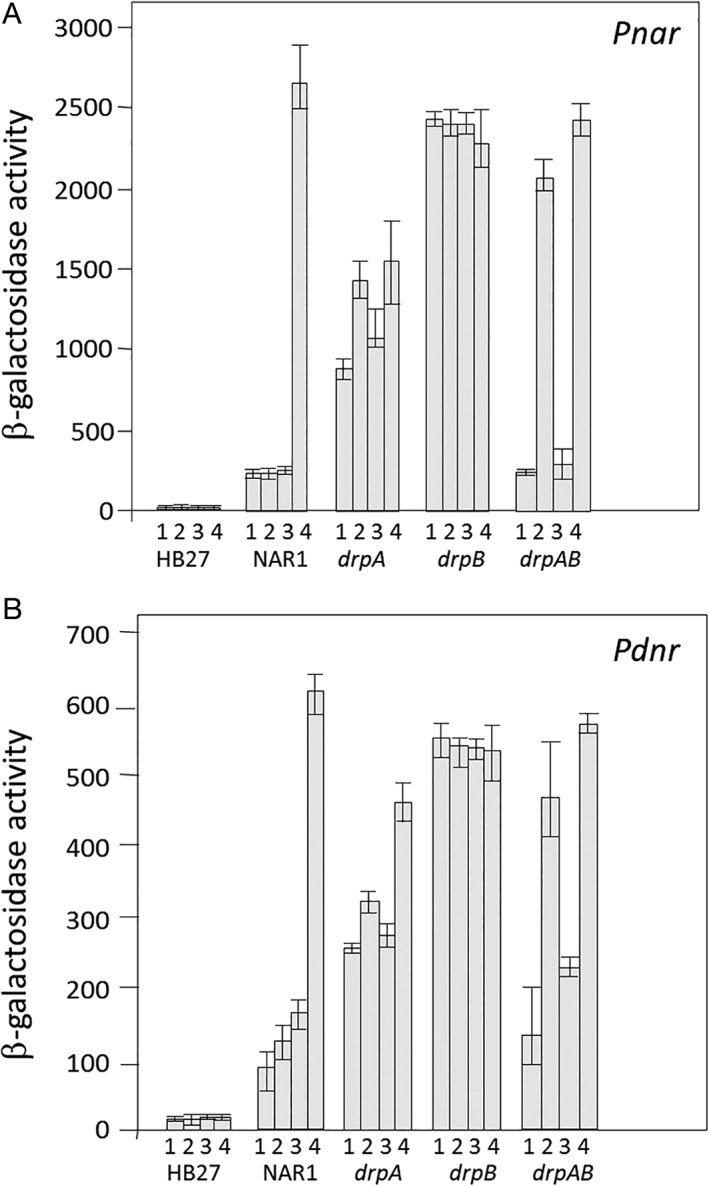
Transcription from the *Pnar* and *Pdnr* promoters in *drp* mutants.Beta‐galactosidase activity expressed from the indicated *Pnar* (A) and *Pdnr* (B) promoters. The activities shown correspond to six samples for each condition from two independent experiments. The organisms assayed were the aerobe HB27, the wild‐type NAR1 *gdh::kat* (NAR1), the *drpA*, *drpB* and *drpAB* mutants. Conditions: (1) aerobic; (2) anaerobic; (3) aerobic plus 20 mM nitrate; and (4) anaerobic plus 20 mM nitrate.

As shown in Fig. 5, the *Pnar* and *Pdnr* promoters were not expressed under any condition in the aerobic strain HB27, showing the requirement for the DnrS and DnrT activators absent from this strain. In the NAR1 strain, basal expression of *Pnar* was detected under aerobic conditions (columns 1 and 3) and under anaerobic conditions without nitrate (column 2) and full induction required anoxia plus nitrate (column 4) as described previously (Cava *et al*., 2007). A similar pattern of expression was detected for *Pdnr*, although at lower levels. In the *drpA* mutant, the two promoters were expressed in all four conditions assayed, with higher levels reached under anoxia, but always below the activity of the wild type in full inducing conditions (compare column 4). In contrast, the absence of DrpB produced more dramatic effects, with apparent constitutive transcription from both promoters under all four conditions. Finally, in the double *ΔdrpAB* mutant both promoters were induced under anoxia (columns 2 and 4) independently of nitrate (absent in column 2). These data corroborated the western blot results of Fig. 4, thus supporting that the effects of *drp* deletion on NarG expression are the consequences of modulation of transcription from its promoter. Also, these data suggest that DrpA/DrpB may affect transcription of the master regulators DnrS and DnrT, meaning that the effects observed on *Pnar* may be indirect.

### *Expression of Pdrp depends on DnrT*

**Figure 6:**
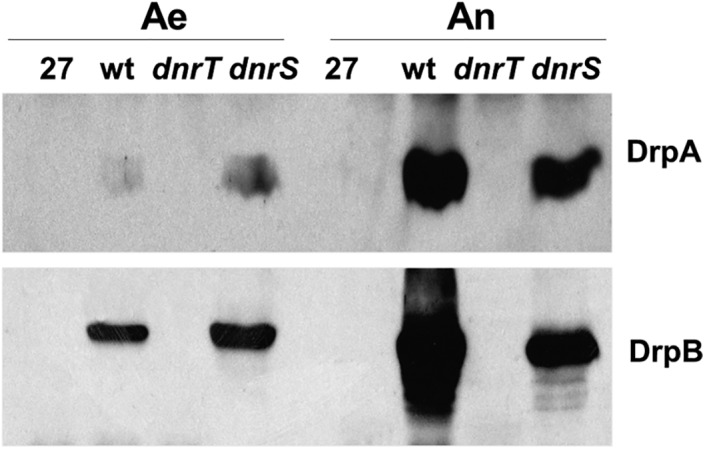
Expression of the *drp* operon depends on DnrT. Western blots to detect the expression of DrpA and DrpB in cultures of the wild‐type NAR1 strain (wt) and corresponding *dnrS* and *dnrT* mutants grown aerobically (Ae) or anaerobically with 20 mM nitrate (An). A control lane with extracts from the aerobic strain HB27 (27) is shown**.**

To determine whether DnrS or DnrT are also required for expression of the *drp* genes, expression of DrpA and DrpB in *dnrS::kat* and *dnrT::kat* mutants was tracked by western blot. Neither DrpA nor DrpB were detected in the *dnrT* mutant under inducing conditions, whereas both proteins were clearly detected in the *dnrS* mutant under the same conditions (Fig. 6). Both DrpA and DrpB were also significantly overexpressed even in aerobic conditions likely as the consequence of constitutive expression of DnrT in *dnrS* mutant (Cava *et al*., 2007). Therefore, expression from the *Pdnr* promoter requires DnrT, but not DnrS.

### *DrpA and DrpB localize to different cell compartments*

**Figure 7:**
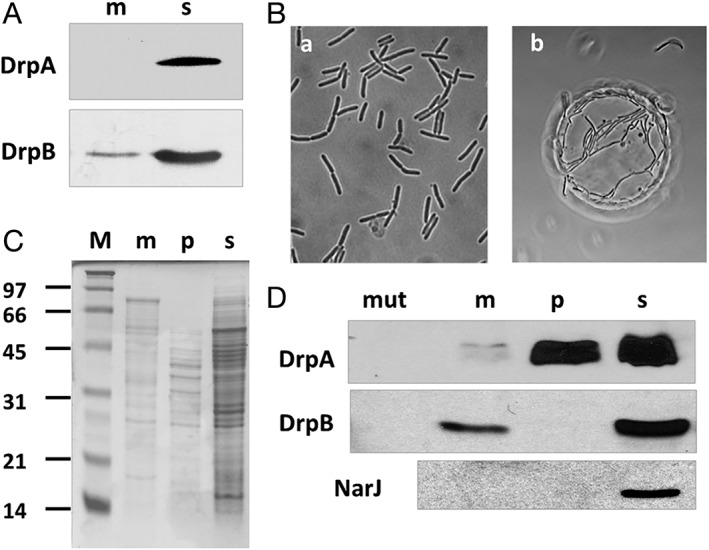
Localization of DrpA and DrpB. A. Western blot to detect DrpA and DrpB in membrane (m) and soluble (s) fractions of T. thermophilus NAR1 grown under anoxic conditions plus nitrate for 4 h. B. Phase‐contrast images of T. thermophilus NAR1 (a) and its *csaB::kat* derivative (b) grown to the end of the exponential phase. C. Cells of the NAR1 *csaB::kat* mutant grown for 16 h under microaerophilic conditions with 20 mM nitrate were harvested and processed to separate the membrane (m), periplasmic (p) and soluble (s) fractions and analysed by SDS‐PAGE and Coomassie blue staining along with molecular weight markers (M).D. The same fractions were analysed by western blot to detect DrpA, DrpB and NarJ. Whole cell extracts from the *drpA* or *drpB* mutants were used as specificity controls for each western blot (lane mut).

Based on the above results and on the analysis of their protein domains, it was hypothesized that DrpA and DrpB may participate in nitrate respiration by signalling the presence of nitrate. Considering that nitrate cannot pass through the cell membrane, one of these proteins should conceivably face the periplasm, although sequence analysis did not indicate the presence of any signal peptide or transmembrane domain. To test the localization of both proteins, we fractionated cells and analysed the different compartments. In a first group of experiments with the wild type strain, all DrpA was found in the soluble fraction (Fig. 7A, s), whereas a part of DrpB appeared to be associated with the membrane (Fig. 7A, m). To further refine this analysis, a *csaB::kat* mutant of the NAR1 strain was isolated. This mutant is defective in pyruvilation of the SCWP (secondary cell wall polymer), which decreases the binding of the outer envelope. As a consequence, they form groups of cells surrounded by a common external membrane, known as multicellular bodies (MBs, Fig. 7B, panel b), from which the periplasmic content can be easily purified by mechanical breakage of the common outer membrane (Castan *et al*., 2002; Cava *et al*., 2004a). Next, expression of DrpA and DrpB proteins was induced in this mutant (nitrate and anoxia conditions) and studied in the periplasmic, membrane and soluble (cytoplasm and periplasm) fractions, which had clearly distinct protein patterns (Fig. 7C). When samples of the three fractions were subjected to western blot, DrpB was absent from the periplasm but detected both in the soluble fraction and, in lesser amounts, associated with the membrane (Fig. 7D). In contrast, DrpA was found basically in the periplasmic and soluble (cytoplasmic and periplasmic from unbroken MBs) fractions, although a small amount was apparently also associated to the membrane fraction. Specific detection of NarJ, a cytoplasmic chaperone required for maturation of the NR (Zafra *et al*., 2005), confirmed the absence of cytoplasmic proteins in the periplasmic fraction, thus discarding cell lysis as the source of DrpA in this compartment. Thus, DrpA and DrpB are expressed in different cell compartments: DrpA is a periplasmic protein that may contact the membrane, whereas DrpB is a cytoplasmic protein that is able to interact with the membrane.

## Discussion

There are no homologues of FNR or the NarX/NarL two‐component system in the genomes of denitrifying strains of *T. thermophilus* that could be responsible for oxygen and nitrate sensing. Previously, the *dnrST* operon was detected upstream of the *nar* operon as the master regulatory element responsible for the expression of all the operons of nitrate and nitrite respiration in this organism (Cava *et al*., 2007; 2008; Alvarez *et al*., 2017). In the regulatory hierarchy, DnrT acts on a concentration‐dependent basis and is insensitive to oxygen and nitrate *in vitro* and *in vivo*, whereas DnrS was proposed to be the oxygen sensor of the system, likely through its N‐terminal GAF domain (Cava *et al*., 2007). However, no hypothesis could be made regarding a putative nitrate sensory system, except that it should be located near the NR operon (which is co‐transferred in conjugative processes) (Alvarez *et al*., 2014a).

**Figure 8:**
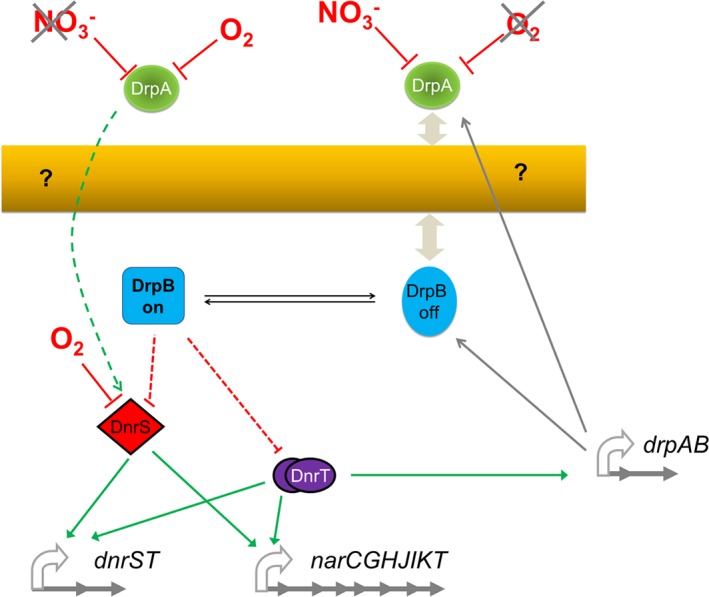
Hypothetical scheme for nitrate control of nitrate respiration in T. thermophilus. Anoxia would be the initial signal for basal expression of the system, with detectable levels of DnrS and DnrT, which would increase expression of the DrpAB system by direct action of DnrT on the Pdrp promoter. DrpA is transported to the periplasm through unknown mechanism (question mark). By default DrpB^on^ form will repress the system, putatively through interaction with DnrS or DnrT (dashed red lines). When nitrate appears in the environment, detection by DrpA would produce a signal that is transduced to DrpB (double arrowheads), leading to its inactive form (DrpB^off^) and allowing for the full expression of the denitrification system. In the absence of DrpB, DnrS and DnrT are overexpressed suggesting additional effects of DrpA on their expression or stability (green dashed line).

The data presented in the current work support that the role of nitrate sensor could be played by the DrpA/DrpB system, encoded immediately downstream of the *nar* operons in all denitrifying *Thermus* spp. so far sequenced (Alvarez *et al*., 2014a). Deletion of the *drpAB* operon rendered induction of the NR and the master operon dependent only on anoxia and, thus, insensitive to the presence of nitrate (Figs 4 and 5), indicating that the system requires these proteins for nitrate sensing. Having in mind our current data, a simple tentative model could be depicted (Fig. 8).

In this model, DrpA stands out as the best candidate for nitrate detection in the periplasm, whereas DrpB would play a role in transducing this signal from the membrane to the transcriptional apparatus. The mechanism by which DrpA reaches the periplasm (dashed arrows in the figure) remains a mystery; no apparent SecB or TAT‐dependent signal peptides could be found at the N‐terminus of the protein, independent of which of the three putative start codons were used. Also, the putative interaction of nitrate with DrpA could not be predicted bioinformatically due to the absence of consensus nitrate binding‐like motifs in its sequence. Moreover, we showed evidence that, similar to the master regulator DnrS (Cava *et al*. 2007), DrpA is not stable in the presence of oxygen (Fig. 4A, lane 3 vs. lane 7), suggesting that absence of oxygen may also be required for nitrate sensing, likely as a security mechanism. Even if DrpA binds nitrate under anaerobic conditions, transduction of the signal to the cytoplasm, where the likely intracellular partner (DrpB) is located, can be only speculated. In this regard, DrpA should be able to contact the external face of the cytoplasmic membrane and the only hint of this is the presence of a faint DrpA band associated with the membrane fraction in western blot assays (Fig. 7).

If such a DrpA‐membrane interaction actually takes place, the next question would regard the molecular nature of the signal transduced to DrpB. There are three domains of unknown function in DrpB, which in other proteins appear associated with sensory hemerytrin domains and also in response regulators. However, no H or D phosphorylation sites were identified in the DrpB sequence and putative Y phosphorylation identified in DrpB by *ad hoc* programs (http://www.hprd.org/PhosphoMotif_finder) is not common in bacteria. Therefore, whatever the transduced signal may be, the system seems unrelated to classical bacterial phosphorylation relays. Thus, a phosphorylation‐independent conformational change or binding/unbinding to the membrane itself may propagate the transduced signal as in other systems (Gaidenko and Price, 2014). These putative two forms (DrpB^On^ and DrpB^Off^) are represented in the figure as quadrangular and oval forms.

Our data support that under noninducing condition, DrpB is expressed at low levels (Figs 2 and 4) in its DrpB^on^ form that somehow represses full transcription of the *Pnar* and *Pdnr* promoters (Fig. 5). Keeping in mind the absence of any detectable DNA binding motif in DrpB (DNAbinder program at http://crdd.osdd.net/raghava/dnabinder/submit.html), such inhibitory effects are likely exerted indirectly, likely via the master regulatory activator proteins DnrS and/or DnrT. In this sense, the DrpB^on^ form may act by inhibiting one of these proteins (red dashed in Fig. 8), limiting their activating functions in transcription either by direct binding or by stimulating its degradation until nitrate is detected outside the cells by DrpA, after which a signalling cascade across the membrane could inactivate or recruit it to the membrane (DrpB^off^), relieving its repressive activity and allowing for the full expression of the system.

However, this model does not fully explain the role of DrpA and DrpB in nitrate respiration. Absence of DrpB in the presence of DrpA produces constitutive expression of the *Pnar* and *Pdnr* promoters independently of the presence of oxygen or nitrate (Figs 4B and 5), whereas absence of both proteins (double Δ*drpAB* mutant) keeps the oxygen control of the system (Figs 4C and 5). Thus, in a *drpB* mutant context, DrpA somehow exerts a signal that keeps the system active. Actually, in agreement with this possibility, the master regulators DnrS and DnrT show and increased concentration in aerobic conditions in this *drpB* ^−^ *drpA* ^+^ strain (Supporting Information Fig. S6).

In addition, it is clear that DrpA and DrpB are acting on additional control levels required for the maturation of the NR activity, as mutants lacking both proteins show little activity despite the wild‐type‐like production of its structural components under anoxia plus nitrate (Fig. 4C). In this sense, it is relevant to note the complexity of this enzyme, of heterotetrameric character, that requires a poorly known membrane‐bound maturation process involving chaperons and a great number of enzymes for the synthesis of its four iron sulfur clusters, two haems b, two haems c and a bis‐(molybdopterin guanine dinucleotide)‐molybdenum cofactor. In this scenario, the possibility exists that the detection of nitrate by the DrpAB system could be required for the expression or functionality of any of these critical pathways, limiting the amount of active NR produced.

## Experimental procedures

### *Strains and growth conditions*

*Thermus thermophilus* HB27 was used as the aerobic control strain. *T. thermophilus* strain NAR1 is a nitrate‐respiring strain (Cava *et al*., 2007), and strain HB27c is a derivative of HB27 that contains the NCE from the NAR1 strain (Alvarez *et al*., 2014b). *Thermus thermophilus* strains VG7, B, CC16, Fiji 3A1, HN1‐11, PRQ16, PRQ15 and NR17 were donated by Dr. M. S. da Costa (University of Coimbra). Aerobic growth was routinely performed on nitrate‐free TB (bacto tryptone 8 g l^−1^, yeast extract 4 g l^−1^, NaCl 3 g l^−1^, pH 7.5 in milliQ water) at 70°C with mild shaking (150 r.p.m.) in a maximum volume of 1/5 flask capacity. Expression of nitrate respiration enzymes was induced after aerobic cultures reached an OD_550_ of 0.3, by stopping the shaker and simultaneously adding 20 mM potassium nitrate. Under these conditions, consumption of O_2_ combined with its low solubility at high temperatures rapidly made the cultures anoxic. Anaerobic cultures with nitrate (20 mM) were carried out in screw‐cap tubes containing 10 ml of TB overlaid with mineral oil (Cava *et al*., 2004). Growth on solid medium was carried out on TB agar (1.5%, wt/vol) plates; kanamycin (Km, 30 mg l^−1^), bleomycin (Bl, 15 mg l^−1^) or hygromycin B (Hy, 50 mg l^−1^) were added to solid and liquid media when required. *Escherichia coli* strains DH5α [*sup*E44, Δ*lac*U169 (ϕ80 *lac*ZΔM15), *hsd*R17, *rec*A, *end*A1, *gyr*A96, *thi‐1*, *relA1*] and BL21(λDE3) [*hsd*S, *gal* (λ*c*Its857, *ind*1, *Sam7*, *nin5*, *lacUV5*‐T7 gene 1)] were used for genetic construction of plasmids and recombinant protein expression, respectively, routinely grown at 37°C on liquid or solid LB medium in the presence of appropriate antibiotics when required.

### *DNA and RNA techniques*

DNA isolation, plasmid purification, restriction analysis, plasmid construction and DNA sequencing were carried out by standard methods (Sambrook *et al*., 1989). PCR was performed with Taq or Pfu DNA polymerases as described by the manufacturer (BIOTOOLS B&M, Madrid, Spain). RNA was isolated using an RNeasy Mini Kit (Qiagen, Hilden, Germany), and after DNAse I treatment (RQ1; Promega, Fitcburg, USA), all samples were tested by conventional PCR to verify the absence of DNA contamination. Reverse transcription was performed using the SuperScript III first strand synthesis kit (Invitrogen, Waltham, USA) according to the manufacturer's instructions. Subsequent PCRs to amplify cDNA were performed using *Pfu* Ultra II Fusion HS DNA Polymerase (Agilent Technologies, Santa Clara, USA) with the appropriate primers.

### *Plasmids and transformation*

The plasmids and primers used are described in Supporting Information Tables S1 and S2 respectively. Plasmids pMHPnarbgaA, pMHPdnrbgaA (Cava *et al*., 2007) and pMHPdrpbgaA are Hy‐selectable derivatives of bifunctional plasmid pMH184 (Cava *et al*., 2007), which contain a thermostable beta‐galactosidase under the control of the *Pnar*, *Pdnr* and *Pdrp* promoters respectively (see Supporting Information for construction details). Plasmids pWURdrpA, pWURdrpB and pWURdrpAB are Bl‐selectable derivatives of pWUR112/77‐1 (Brouns *et al*., 2005) used for the constitutive expression of DrpA, DrpB and both proteins respectively. Their construction is described in the Supporting Information. Transformation of *T. thermophilus* with linear or circular DNA was achieved by natural competence (Koyama *et al*., 1986), whereas standard protocols were used to transform *E. coli* (Hanahan, 1985).

### *Isolation of mutants and complementation assays*

Construction of knockout mutants was carried out by insertion of a *kat* gene cassette conferring thermostable resistance to Km (Lasa *et al*., 1992). For this, we used a derivative of pUC19 (pDOWN1) that contains the final region of the *nar* operon and downstream regions, including the *drp* operon. Suicide constructions were obtained by insertion of the *kat* gene cassette into selected restriction sites of this plasmid (Supporting Information Table S1) and linearized before transformation to force double recombination. Due to the higher transformation efficiency of the HB27 strain compared to NAR1, strain HB27c was first transformed with linear DNA and mutations were subsequently transferred to the NAR1 strain by transformation using genomic DNA from the HB27c mutants. Km‐resistant clones were selected and analysed by PCR to confirm *kat* insertion and by western blot to ensure the absence of the targeted protein. Construction of deletion mutants *dnrS::kat* and *dnrT::kat* was previously described (Cava *et al*., 2007).

### *Production and detection of recombinant proteins*

For the recombinant overexpression of DrpA and DrpB, each gene was amplified with the primers indicated in Supporting Information Table S2, incorporating restriction sites for NdeI and EcoRI. Amplified fragments were cloned into the equivalent restriction sites of pET22b, and expression of the respective constructs was carried out in *E. coli* BL21(λDE3) by adding 1 mM IPTG (β*‐* d‐1‐thiogalactopyranoside) to exponential cultures grown at 37°C to OD_550_ = 0.5. Cells were harvested after approximately 4 h at 37°C, cooled to 4°C, disrupted by sonication (two 30‐s pulses at 18 μm of amplitude, at 0.5‐s intervals with a LABSONIC U‐Braun) and centrifuged (2,000*g*, 10 min, 4°C) to eliminate cell debris and insoluble cell components. The supernatant was heated to 70°C for 30 min to denature the host proteins, and recombinant thermostable soluble proteins were obtained upon centrifugation (20,000*g*, 17 min, 4°C). The soluble proteins were separated in a SDS‐PAGE gel. The gel fragments containing pure DrpA or DrpB proteins were used to immunize New Zealand rabbits, whose sera were collected and used as antibodies to detect proteins by standard luminescent western blot procedures.

### *Cell fractionation*

*Thermus thermophilus* mutants lacking the CsaB protein have defects in attachment of the outer membrane to the underlying peptidoglycan layer, producing MBs with a fragile common outer membrane and a huge periplasmic space (Cava *et al*., 2004a). The *csaB::kat* mutant of *T. thermophilus* NAR1 was obtained by transformation of the wild‐type (wt) strain with chromosomal DNA from the HB27 *csaB::kat* derivative (Cava *et al*., 2004a). The NAR1 wt and its *csaB::kat* derivative were incubated with nitrate (20 mM) under anoxic conditions and further processed in different ways. Wt cells were harvested by centrifugation, resuspended in buffer A (Tris–HCl 50 mM, NaCl 50 mM, pH 7.5) containing a cocktail of protease inhibitors (Complete mini‐Roche) and broken by sonication as earlier. After discarding unbroken cells (2,000*g*, 3 min, 4°C), the samples were centrifuged (20,000*g*, 30 min, 4°C) to separate membrane and soluble fractions. Membranes were re‐suspended in a volume of buffer A equivalent to that of the soluble fraction, and identical volumes of both fraction samples were separated by SDS‐PAGE (10% acrylamide) and subjected to western blot for the detection of DrpA and DrpB. Processing of the *csaB::kat* mutants required mild harvesting of the cells (3,000*g*, 5 min) followed by mechanical breaking of MBs by repeated pipetting of the cell pellets (Cava *et al*., 2004a). The soluble fraction obtained by centrifugation (20,000*g*, 30 min, 4°C) was defined as the periplasm, whereas the cell pellet was further processed as described earlier for wt cells.

### *Promoter induction assays and NR activity*

Quantitative measurements of the transcription from the *Pnar*, *Pdrp* and *Pdnr* operons were made in *T. thermophilus* cultures of strains transformed with pMHPnarbgaA, pMHPdrpbgaA and pMHPdnrbgaA respectively. The β‐galactosidase activities of soluble cell extracts of six individual colonies from duplicate experiments were assayed at 70°C with the chromogenic substrate ortho‐nitrophenyl‐β‐d‐galacto‐pyranoside (ONPG) (Cava *et al*., 2007) and expressed as described by Miller (1992).

NR activity was measured at 80°C with reduced methyl‐viologen (MV 1,1′‐dimetthyl‐4,4′‐bipiridinium) as an electron donor and 20 mM potassium nitrate as an electron acceptor (Ramírez‐Arcos *et al*., 1998a). One enzyme unit was defined as the enzyme amount required to produce 1 nmol of nitrite per minute. Total units were normalized to 1 ml of a culture at OD_550_ = 1 (~ 10^9^ cells).

### *Sequencing and bioinformatics*

Constructs were sequenced by the dideoxy‐nucleotide method. Sequence analysis was carried out online with the BLASTP 2.2.19 program (Altschul *et al*., 1997). The domain structure of the proteins was analysed using the InterPro repository (Finn *et al*., 2017). The sequence of the *drpAB* cluster of the NAR1 strain was deposited in GenBank (accession number MH037153).

## Supporting information


**Appendix S1.** Supporting Information
