# Supplementary material for: The role of conserved proteins DrpA and DrpB in nitrate respiration of Thermus thermophilus
Source: Environ Microbiol. 2018 Oct 2;20(10):3851–61. doi: 10.1111/1462-2920.14400 (PMC6282519; doi:10.1111/1462-2920.14400)
Supplement: Supplementary file 1 — Appendix S1. Supporting Information [file EMI-20-3851-s001.doc]

Revised version 2

SUPPORTING INFORMATION TO:

**The role of conserved proteins DrpA and DrpB in nitrate respiration of *Thermus thermophilus***

**Zahra Chahlafi^1^, Felipe Cava^2^, Laura Alvarez^2^ and José Berenguer^1^**

^1^ *Centro de Biología Molecular Severo Ochoa. Universidad Autónoma de Madrid-Consejo Superior de Investigaciones Científicas. Madrid, 28049, Spain*

^2^ *Department of Molecular Biology. Umeå University SE-901 87 Umeå. Sweden*

**Figure S1. Chahlafi, Z et al. 2018**

| 1 | | GATCTTTCCGCCCCGCCAGGCGGGGGGAGTTATCGGCTGGACCGCGGCCGTCGCCGCCTACGGGCCTTTCCTCTTCTCCACCCTGGCTGC |  |
| --- | --- | --- | --- |
| 91 | | I F P P R Q A G G V I G W T A A V A A Y G P F L F S T L A A  CTATACCCAGCAGGCCACGGGAGGCTTCACCGCCTTCTTCTACGGGCTCATGGTCTTCTACGCCTTCAACTTCTTCCTGAACTGGTACTA |  |
| 181 | | Y T Q Q A T G G F T A F F Y G L M V F Y A F N F F L N W Y Y  CTACGCCCGCAAGGGGGCGGAGAAGCCCTGCTAAGTACCCCACCGCGGCAAAAGCCGCGGTGGGGGCCCCAAAAGGAGGTGCCCATGCTC  Y A R K G A E K P C * M L | 2 |
| 271 | | CGGGCGGGGGAGCCCCGCCCGGGGCTTTCCTTGACCCAGGTCATGGCCCAGGCCCTCGGGCCTAGGGAAACTGAAGGCGCTATGGAGCTG |  |
|  | | R A G E P R P G L S L T Q V M A Q A L G P R E T E G A M E L | 32 |
|  | | *Bgl*II |  |
| 361 | | GACGTGCGCGCGATCCCCCCTAGGGAAAGGCACCCCAAGATCTTCGGCGTCTTTGACGCCCTGGCCCCCGGCGAGAGCTTCGTCCTCGTC |  |
|  | | D V R A I P P R E R **H** P K I F G V F D A L A P G E S F V L V | 62 |
| 451 | | AACGACCACGACCCCAAGCCCCTCTACTACCAGCTCATGGCTGAGCGCCCGGGCCAGGTGGACTGGACCTATTTGGAGGAGGGTCCGGAG |  |
|  | | N D **H** D P K P L Y Y Q L M A E R P G Q V D W T Y L E E G P E | 92 |
|  | | *Xho*I |  |
| 541 | | GTATGGCGGGTGCGGATCGGGAAGAAGTAGTCCGCCCCGAGATGAAGGTGGCGGAGGTCCTAAAGCGCTGGCCCCACCTCCTCGAGGTCC |  |
|  | | V W R V R I G K K * | 101 |
|  | | M A G A D R E E V V R P E M K V A E V L K R W P H L L E V L | 29 |
| 631 | | TGGTGGAGGCCAGCCCCGCCTTCCAGAAGCTCAAAAACCCCCTCCTCCGCCGCACCATGCCCAACCTGGTCACCGTGGCCCAGGCGGCGA |  |
|  | | V E A S P A F Q K L K N P L L R R T M P N L V T V A Q A A R | 59 |
| 721 | | GGATCGGAGGCCTGGAGCCGGAGGAGCTCGTGGCCAGGCTCAACCGGGCCCTGGGGGTGGAGGCCAGGCCCCCCGTGGCCAAGGCGGAGG |  |
|  | | I G G L E P E E L V A R L N R A L G V E A R P P V A K A E E | 89 |
| 811 | | AGAGCCTCCTCGGCACCCCGCCCCCTCCCTGGCTTTCCGCCCCCCTAGGCTTCCACCTGGACGTCCGGCCCATCCTGGAAGGAGGTGGGG |  |
|  | | S L L G T P P P P W L S A P L G F H L D V R P I L E G G G E | 119 |
| 901 | | AGCCTTTCCAAGCCATCATGGCGGCGGCCAAGGAGGTGAAGCCCGGGGAGAGGCTCGTGCTGGAGGTGCTCTTTGAGCCCGTGCCCCTCT |  |
|  | | P F Q A I M A A A K E V K P G E R L V L E V L F E P V P L Y | 149 |
| 991 | | ACCGGGTCCTGGCCAAGCAGGGCTTTCTCGCCTGGTGCGAGAAGGTGGGGGAGCGGCACTACCGGGCCCACTTCTACCGACAGGAGGTGG |  |
|  | | R V L A K Q G F L A W C E K V G E R H Y R A H^*^ F Y R Q E V G | 179 |
| 1081 | | GGGAGGGGAGGAAAGGGGAAAGGGACCATGCCGCCCCCCTCACCGAGGCGGACTGGGAGGACTTCCAGGCCGAGGTCTTCATTGAGGAGA |  |
|  | E G R K G E R D H A A P L T E A D W E D F Q A E V F I E E N | | 209 |
| 1171 | | ACCTGGAGCCCCCCTTGCCCATGATGCGGGTGCTGGAGGCCTTGGCCAGGCTAAGGCCCGGGGAGAAGCTTCTCGTCCACCACGTGCGCA |  |
|  | | L E P P L P M M R V L E A L A R L R P G E K L L V H H V R R | 239 |
|  | | *Xho*I |  |
| 1261 | | GGCCCATCCACCTCCTCGCCCGCCTCGAGGAGGAGGGGCACGCTTACCTGCTCAAGGACCTGGGCCCGGGGCAGGTGAAGATCCTGATCC |  |
|  | | P I H L L A R L E E E G H A Y L L K D L G P G Q V K I L I R | 269 |
| 1351 | | GGAAAGGGGGGTAGGCCCTCAAAGCCCATGGGCTTTTGGCACTTCCTCTTCATCCGGGCGGCTCTTTTATACCTTCGTTTGCTACTTGA |  |
|  | | K G G * | 273 |

**Figure S1. Sequence of the *drp* operon.** Sequence of the regions downstream of the *narGHJIKT* operon from *T. thermophilus* NAR1. Sequences in Blue correspond to the C-terminus of NarT; Sequences in Red correspond to DrpA, with the likely starting methionine underlined; Sequences in Green correspond to DrpB. Restriction sites for BglII and XhoI used to get the corresponding insertion mutants are labelled above the sequence. Inverted repeats that could correspond to transcription terminator structures are underlined.

**Figure S2. Chahlafi, Z et al. 2018**

DrpA -----------------------MELDVRAIPPRER**H**PKIFGVFDALAPGESFVLVND**H**DPKPLYYQLMAERPGQVDWTYLEEGPEVWRVRIGKK---

[WP_014511190.1](https://www.ncbi.nlm.nih.gov/protein/WP_014511190.1?report=genbank&log$=prottop&blast_rank=1&RID=9JVRN2AW211) -----------------------MELDVRAIPPRER**H**PKIFGVFDALAPGESFVLVND**H**DPKPLYYQLMAERPGQVDWTYLEEGPEVWRVRIGKK---

[WP_093007219.1](https://www.ncbi.nlm.nih.gov/protein/WP_093007219.1?report=genbank&log$=prottop&blast_rank=2&RID=9JVRN2AW211) -----------------------MELDVRAIPPRER**H**PKIFGVFDALAPGESFVLVND**H**DPKPLYYQLLAERPGQVDWTYLEEGPEVWRVRIGKK---

[WP_018112083.1](https://www.ncbi.nlm.nih.gov/protein/WP_018112083.1?report=genbank&log$=prottop&blast_rank=3&RID=9JVRN2AW211) -----------------------MELDVRTIPPRER**H**PRIFGVFDSLKPGQSFVLVND**H**DPKPLYYQLMAERPGQVDWTYLEEGPEVWRVRIGKK---

[ADW22359.1](https://www.ncbi.nlm.nih.gov/protein/ADW22359.1?report=genbank&log$=prottop&blast_rank=4&RID=9JVRN2AW211) -----------MADGEKAGEAWAMELDVRTLPPRER**H**PRIFALFDALKPGESFLLVND**H**DPKPLYYQLMAERPGQVDWTYLEEGPEVWRVRIGKR---

[ETN87529.1](https://www.ncbi.nlm.nih.gov/protein/ETN87529.1?report=genbank&log$=prottop&blast_rank=5&RID=9JVRN2AW211) -----------MADGEKAGEAWAMELDVRTLPPRER**H**PRIFALFDALKPGESFLLVND**H**DPKPLYYQLMAERPGQVDWAYLEEGPEVWRVRIGKR---

[WP_028493912.1](https://www.ncbi.nlm.nih.gov/protein/WP_028493912.1?report=genbank&log$=prottop&blast_rank=6&RID=9JVRN2AW211) -----------------------MELDVRTLPPRER**H**PRIFALFDALKPGESFVLVND**H**DPKPLYYQLMAERPGQVDWAYLEEGPEVWRVRIGKR---

[WP_038043459.1](https://www.ncbi.nlm.nih.gov/protein/WP_038043459.1?report=genbank&log$=prottop&blast_rank=7&RID=9JVRN2AW211) -----------------------MELDVRTLPPRER**H**PRIFALFDGLKPGESFVLVND**H**DPKPLYYQLMAERPGQVDWVYLEEGPEVWRVRIGKR---

[WP_038057438.1](https://www.ncbi.nlm.nih.gov/protein/WP_038057438.1?report=genbank&log$=prottop&blast_rank=8&RID=9JVRN2AW211) -----------------------MELDVRTLPPRER**H**PRIFALFDSLKPGESLVLVND**H**DPKPLYYQLMAERPGQVDWAYLEEGPEVWRVRIGKR---

[WP_016329275.1](https://www.ncbi.nlm.nih.gov/protein/WP_016329275.1?report=genbank&log$=prottop&blast_rank=9&RID=9JVRN2AW211) -----------------------MELDVREIPPRER**H**PRIFALFDALKPGEGFVLVND**H**DPKPLYYQFQAERPGQVDWTYLEEGPSVWRVRIGKR---

[WP_071676453.1](https://www.ncbi.nlm.nih.gov/protein/WP_071676453.1?report=genbank&log$=prottop&blast_rank=10&RID=9JVRN2AW211) -----------------------MELDVRTIPPRER**H**PRIFGVFDSLKPGQSFVLVND**H**DPRPLYYQFQAERPGAFAWEYLEAGPEVWRVRITRQ---

[WP_013156815.1](https://www.ncbi.nlm.nih.gov/protein/WP_013156815.1?report=genbank&log$=prottop&blast_rank=11&RID=9JVRN2AW211) MTQVMAKKAAPPHTEAEVNFMPIQILDVRSIPPRER**H**PRIFHLFDSLGDGQAFELVND**H**DPKPLFYQFSAERPEQFGWEYLEQGPETWRVRIRKK--G

[OOP54817.1](https://www.ncbi.nlm.nih.gov/protein/OOP54817.1?report=genbank&log$=prottop&blast_rank=12&RID=9JVRN2AW211) -----------MS------KTPSVVLDVRSIVPRER**H**PKIFNTFDGLKKGEMMVLIND**H**DPKPLKYQLDAERSGQLDWKYVEQGPEVWKVEITKK---

[KXB97269.1](https://www.ncbi.nlm.nih.gov/protein/KXB97269.1?report=genbank&log$=prottop&blast_rank=13&RID=9JVRN2AW211) ----MLLERVHSHPRPQPPAAGERELDVRAIPPAQR**H**PLIFATFDQLPNGGWFVLVND**H**DPKPLYYQFQFERSGQLDWEYLESGPEVWRVRITKRSEG

**** * *** ** ** * * * ***** ** ** ** * * * ** * * *

**Figure S2. Alignment of DrpA.** Figure shows the alignment of the DrpA protein with the following proteins of the gene bank: [WP_014511190.1](https://www.ncbi.nlm.nih.gov/protein/WP_014511190.1?report=genbank&log$=prottop&blast_rank=1&RID=9JVRN2AW211) (*Thermus thermophilus*); [WP_093007219.1](https://www.ncbi.nlm.nih.gov/protein/WP_093007219.1?report=genbank&log$=prottop&blast_rank=2&RID=9JVRN2AW211) (*Thermus arciformis*); [WP_018112083.1](https://www.ncbi.nlm.nih.gov/protein/WP_018112083.1?report=genbank&log$=prottop&blast_rank=3&RID=9JVRN2AW211) (*Thermus igniterrae*); [ADW22359.1](https://www.ncbi.nlm.nih.gov/protein/ADW22359.1?report=genbank&log$=prottop&blast_rank=4&RID=9JVRN2AW211) (*Thermus scotoductus* SA-01); [ETN87529.1](https://www.ncbi.nlm.nih.gov/protein/ETN87529.1?report=genbank&log$=prottop&blast_rank=5&RID=9JVRN2AW211) (*Thermus sp.* NMX2.A1); [WP_028493912.1](https://www.ncbi.nlm.nih.gov/protein/WP_028493912.1?report=genbank&log$=prottop&blast_rank=6&RID=9JVRN2AW211) (*Thermus antranikianii*); [WP_038043459.1](https://www.ncbi.nlm.nih.gov/protein/WP_038043459.1?report=genbank&log$=prottop&blast_rank=7&RID=9JVRN2AW211) (*Thermus tengchongensis*); [WP_038057438.1](https://www.ncbi.nlm.nih.gov/protein/WP_038057438.1?report=genbank&log$=prottop&blast_rank=8&RID=9JVRN2AW211) (*Thermus amyloliquefaciens*); [WP_016329275.1](https://www.ncbi.nlm.nih.gov/protein/WP_016329275.1?report=genbank&log$=prottop&blast_rank=9&RID=9JVRN2AW211) (*Thermus oshimai*); [WP_071676453.1](https://www.ncbi.nlm.nih.gov/protein/WP_071676453.1?report=genbank&log$=prottop&blast_rank=10&RID=9JVRN2AW211) (*Thermus brockianus*); [WP_013156815.1](https://www.ncbi.nlm.nih.gov/protein/WP_013156815.1?report=genbank&log$=prottop&blast_rank=11&RID=9JVRN2AW211) (*Meiothermus silvanus*); [OOP54817.1](https://www.ncbi.nlm.nih.gov/protein/OOP54817.1?report=genbank&log$=prottop&blast_rank=12&RID=9JVRN2AW211) (Candidatus *Brocadia caroliniensis*); [KXB97269.1](https://www.ncbi.nlm.nih.gov/protein/KXB97269.1?report=genbank&log$=prottop&blast_rank=13&RID=9JVRN2AW211) (*Chlorobi* bacterium NICIL-2). Conserved Histidines are labeled in red.

**Figure S3. Chahlafi, Z et al. 2018**

**DrpB**  MAGADREEVVRPEMKVAEVLKRWPHLLEVLVEASPAFQKLKNPLLRRTMPNLVTVAQAARIGGLEPEELVARLNRALGVE 80

**WP_026175165**  MAGADREEVVRPEMKVAEVLKRWPHLLEVLVAASPAFQKLKNPLLRRTMPNLVTVAQAARIGGLEPEELVARLNRALGVE 80

**WP_014511189**  MAGADREEVVRPEMKVAEVLKRWPHLLEVLVAASPAFQKLKNPLLRRTMPNLVTVAQAARIGGLEPEELVARLNRALGVE 80

**WP_093007221**  MAGADREEVVRPEMKVAEVLKRWPHLLEVLVAASPAFQKLKNPLLRRTMPNLVTVAQAARIGGLTPEELVARLNRALGVE 80

**WP_028493913**  MAGEDRQEVVRPEMKVAEVLRRWPELLEVLAEASPAFQKLKNPLLRRTMPNLVTVAQAARIGGLEPEELVARLNRALGVE 80

**WP_038032297**  MAGEDRQEVVRPEMKVAEVLRRWPELLEVLAEASPAFQKLKNPLLRRTMPNLVTVAQAARIGGLEPEELVARLNRALGVE 80

**WP_038041980**  MAGEDRQEVVRPEMRVAEVLRRWPELLRVLVEASPAFQRLKNPLLRKTLPNLVTVAQAARMGGLEPEELVARLNRALGVE 80

**WP_019550645**  MAGADREEVVHPGMKVAEVLARWPHLLEVLVAASPAFQKLKNPLLRRTMPALVTVAQAARIGGLEPEELVARLNRALGVE 80

**WP_038057436**  MAGEDRQAVVHPEMKVAEVLRRWPELLQVLVEASPVFQKLKNPLLRKTMPNLVTVAQAARMGGLEPEELVARLNRALGVE 80

**WP_026234264**  MAGKDREKVT-PEMKVGEVLRRWPHLLEVFLEASPAFQKLKNPLLRRTMPNLVTVAQAERMGGLEPGTLVARLNRALGVE 79

**APD08618**  --------MVRPEMKVAEVLKRWPGLLEVFLEASPAFQKLKNPLLRRTMSNLVTVAQAARVAGLAPEELVARLNQALGVA 72

**AEB12277**  --------MVRPEMKVSEVLQRWPELLEVLVEASPAFRRLHNPLLRRTMPRLVTVAQAARIAGMPPETLVSQLNAALGVE 72

**WP_018467766**  --------MIRPEMRVSELLEAHPQLLGVLIEASPAFAKLKNPLLRRTMPRLVTLAQAARIGGLEPGALIERLNRALGLE 72

* * * * * * ** * ****** ******* ** *** ***** * * * ** **

**DrpB**  ARP---PVAKAEESLLGTPPPPWLSAPLGFHLDVRPILEGGGEPFQAIMAAAKEVKPGERLVLEVLFEPVPLYRVLAKQG 157

**WP_026175165** ARP---PVAKAEESLLGTPPPPWLSAPVGFRLDVRPTLEAGGEPFQAIMAAAKEVKPGERLVLEVLFEPIPLYKVLGKQG 157

**WP_014511189** ARP---PVAKAEESLLGAPPPPWLSAPLGFHLDVRPILEGGGEPFQNIMAAAKEVAPGERLVLEVLFEPVPLYRVLAKQG 157

**WP_093007221** ARL---PVAGAEESLLGAPPPPWLSAPVGFHLDVRPILEGGGEPFPNIMAAAKEVAPGERLVLEVLFEPVPLYRVLAKQG 157

**WP_028493913** ARP-EVPVAKEGESLLGTPPPPWLSAPVGFHLDVRPILEQGGEPFQAIMAAAREVGPGTKLVLEVLFEPIPLYKVLGKQG 159

**WP_038032297** ARP-EVPVAKEGESLLGTPPPPWLSAPVGFHLDVRPMLEQGGEPFQAIMAAAREVGPGTKLVLEVLFEPIPLYKVLGKQG 159

**WP_038041980** ARP-EV-LGGEAESLLGAPPPPWLSAPLGFHLDVRPILERGGEPFQAIMAAAREVGPGERLVLEVLFEPIPLYKVLGKQG 158

**WP_019550645** AGP-AFPVA--EESLVGTPPPSWLSAPVGFRLDVRPTLEAGGEPFSRILAAAREVKPGERLVLEVLFEPIPLYRVLAKQG 157

**WP_038057436** ARP-EVPIG-QAESLLGTPPPSWLSAPVGFQLDVRPILERGGEPFQAIMAAAQEVEPGKRLVLEVLFEPIPLYKVLGKQG 158

**WP_026234264** ELP-QAPSG-EGESLLQAPPPSWLSAPLGFHLDVRPILEAGGEPFQAIMAAAKEVKPGERLVLEVLFEPIPLYKVLAKQG 157

**APD08618**  SEA-PPPSS-LEESLLLTPPPPWLDAPVGFHLDVRPILERGGEPFAHIMAAAKEVGPGKRLVLEVLFEPVPLYRVLGKQG 150

**AEB12277**  TKPMEMPIA--SDSKLHTPPPPWLQTPVGAHLDVRPILEAGEEPFPKIMAAARTVKPGGRLVLEAPFEPLPLYRVLAKQG 150

**WP_018467766** VAGEKASVG--NESKLGTPPPDWLAAPVGFHLDVRPILAAGGEPFGAIMAAAREVAAGQRLCLEAPFEPLPLYRVMQRQG 150

* *** ** * * ***** * * *** * *** * ** ** *** *** * **

**DrpB**  FLAWCEKVGERHYRAHFYRQEVGEGRKGERDHHAAPLTEADWEDFQAEVFIEENLEPPLPMMRVLEALARLRPGEKLLVH 237

**WP_026175165** FLAWCERLGERHYRAHFYRQEVGEGRKGERDHA-APLTEADWEDFQAEVFIEENLEPPLPMMRVLEALAALKPGEKLLVH 236

**WP_014511189** FLAWCEKVGERHYRAHFYRQGAGEGRKGGKGPA-APLTEADWGDYQAEVFIEENLEPPLPMMRVLEALAALKPGEKLLVH 236

**WP_093007221** FLAWCEKVGERHYRAHFYRQGAGEG---GRGPA-VPLTEADWEDYQAEVFIEENLEPPLPMMRVLEALAALKPGERLLVH 233

**WP_028493913** FLAWCERLGERHYRVHFYRQEVRQGQ-GVAAGP-ASLSEEDWRDYQAEVFIEENLEPPLPMMRVLEALARLRPGEKLLVH 237

**WP_038032297** FLAWCERLGERHYRVHFYRQEVRQGQ-GVAAGP-ASLSEEDWRDYQAEVFIEENLEPPLPMMRVLEALAGLRPGEKLLVH 237

**WP_038041980** FLAWCERLGERHYRVHFYRQGVGKGQ-EVAAGP-ASLSEADWESYQAEVYIEENLEPPLPMMRVLEALAGLKPGEKLLVH 236

**WP_019550645** FLAWCEKVGERHYRAHFYRQ--GTAAEKGLAKA-PPLTEADWEEVQAEVTIEENLEPPLPMMRVLEALAALKPGEKLLVH 234

**WP_038057436** FLAWCERLGERHYRVHFYRQQFGEGR-GVAAGP-GPLSEGDWEDYQAEVQIEENLEPPLPMMRVLEALAGLKPGEKLLVH 236

**WP_026234264** FLAWCERVGERHYRAHFYRK--GQGEAGVPRE-APFTEEAWANPHKEVTIEPHWEPPLPMQAVL-EALAELKPGQRLLVH 234

**APD08618**  FAAWCEKLGENHFRAHFYRESVGESQ---QAPPlRSLAEEDWEDYQAEVVVEENLEPPLPMVRVLEALASLRPGEKLLVH 227

**AEB12277**  FSAWCEQLGAQHYRVHFYREPLPTQPEPTSSSS-EPLTEADWQAHHAELTVDASWEPPLPMQRILEALAALEPGQRLLVH 229

**WP_018467766** FVAWCEQLGPEHYRVHFLREREQEAK----GRAARTLGEADWASYAAEVTIEANLEPPLPMMRVLEALARLEPGQRLRVH 226

* **** * * * ** * *** **** * ** * **

**DrpB**  HVRRPIHLLARLEEEGHAYLLKDLGPGQVKILIRKGG 274

**WP_026175165** HVRRPIHLLARLEEEGHAYLLKDLGPGQVKILIRKGG 273

**WP_014511189** HVRRPIHLLARLEEEGHAYALKDLGPGRVELLIRKGG 273

**WP_093007221** HVRRPIHLLARLEEEGHAYALKDLGPGRVEILIRKGG 270

**WP_028493913** HVRRPVHLLARLEEEGHAYLLKDLGPGQVKILIRKGG 274

**WP_038032297** HVRRPVHLLARLEEEGHAYLLEDLGPGQVKILIRKGG 274

**WP_038041980** HVRRPVHLLARLQAEGHTYLLKDLGPGQVKILIRKGG 273

**WP_019550645** HVRRPIHLLARLEEEGHAYALKDLGPGKVKLLIRKGG 271

**WP_038057436** HVRRPVHLLARLEEEGHAYLLKDLGPGRVEILIRKGG 273

**WP_026234264** HVRRPIHLLARLEEEGVRYALKDLGPGRVEILIEKP- 270

**APD08618**  HVRRPIHLLARLEEEGHAYALKDLGPGQVKLLIRKGG 264

**AEB12277**  HVRRPIHLLARLEAEGYRYALRDLGPGRVDLLIEKPR 266

**WP_018467766** HVRRPVHLLARLAQDGHPYALRELGPGQVELLIEKRS 263

***** ****** * * * **** * ** *

**Figure S3. Alignment of DrpB sequence.** Figure shows the alignment of DrpB with the following proteins: WP_026175165.1 [*Thermus igniterrae*]; WP_014511189.1 [*Thermus thermophilus*]; WP_093007221.1 [*Thermus arciformis*]; WP_028493913.1 [*Thermus antranikianii*]; WP_038032297.1 [*Thermus sp.* NMX2.A1];  WP_038041980.1 [*Thermus tengchongensis*]; WP_019550645.1 [*Thermus scotoductus*]; WP_038057436.1 [*Thermus amyloliquefaciens*]; WP_026234264.1 [*Thermus oshimai*]; APD08618.1 [*Thermus brockianus*]; AEB12277.1 [*Marinithermus hydrothermalis* DSM 14884]; WP_018467766.1 [*Meiothermus* timidus]

**Figure S4. Chahlafi, Z et al. 2018**

**
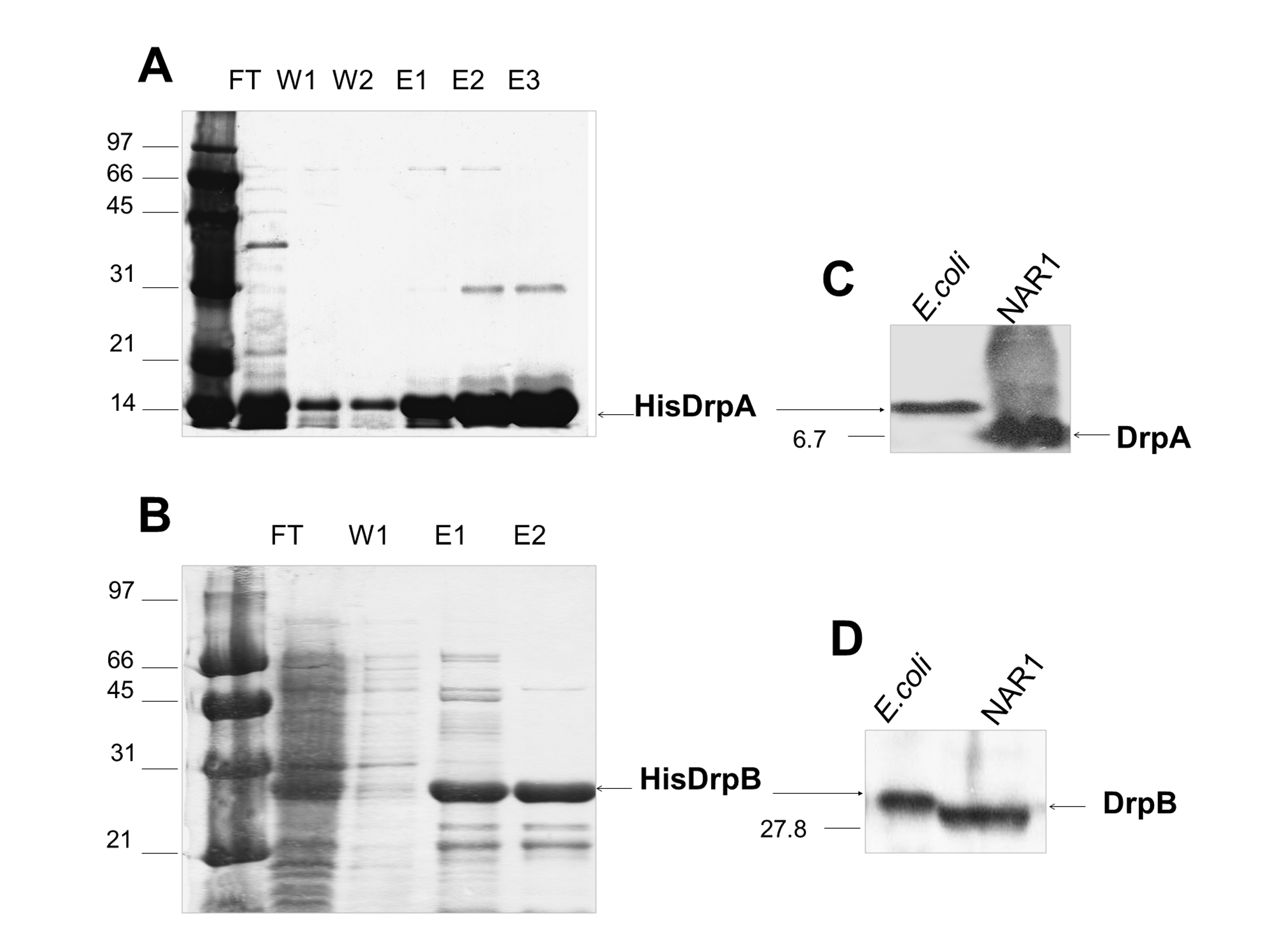
**

**Figure S4. Recombinant expression of Drp proteins.** Coomassie-blue stained SDS-PAGE showing the over-expression in *E. coli* and purification of DrpA (A) and DrpB (B). FT: soluble fraction from overexpressing cultures after incubation at 70 ºC for 15 min; W1, W2) Proteins eluted after passing through the FT fraction through to the Ni-agarose support; E1-E3) Fractions eluted with imidazole 150 mM. Western blots with antisera raised against DrpA (C) and DrpB (D) showing the expression of the corresponding proteins in the *T. thermophilus* NAR1 strain compared with the respective His-tagged purified protein produced in *E.coli.* Experimentally calculated protein sizes are indicated for the native proteins.

**Figure S5. Chahlafi, Z et al. 2018**

**Figure S5. Isolation of *drp* mutants**. A) Scheme of the genetic structure of the wild type and its *drp* mutant derivatives. The genes *narT* (nitrite/nitrate transporter) and *tnp* (putative transposase) are also indicated. B-D) PCR assays to detect the presence of the *kat* gene in different clones (left) and western blot to detect the products of the DrpA and DrpB proteins (right) in the *drpA* (A), *drpB* (B) and *drpAB* (D) mutants. Lanes A1-3, B1-3 and AB1-2 indicate individual *drpA::kat*, *drpB::kat* and *drpAB::kat* mutants derived from the NAR1 (NAR) strain. The aerobic HB27 strain is used as negative control for the western blots.

**Figure S6. Chahlafi, Z et al. 2018**

**Figure S6. Effect of the absence of DrpA or DrpB in the expression of the Dnr master regulator proteins.** Cultures of the wild type NAR1 strain (Wt) and the *drpB* (ΔB) mutant transformed with an empty pWUR plasmid, and the *drpB* mutant transformed with a pWUR derivative that overexpresses DrpB (+B) were grown under aerobic conditions without nitrate (Ae) or incubated for 12 h under anaerobic conditions with nitrate (An). Proteins DnrS and DnrT were detected by western blot.

**Table S1**. Plasmids used in this work.

| **NAME** | **USE/PROPERTIES** | **REFERENCE** |
| --- | --- | --- |
| pUC119 | Cloning vector for *E. coli*, Am | (Vieira and Messing, 1982) |
| pET28b | Expression vector for *E. coli*, Km | Novagen |
| pMH184 | Cloning shuttle *E. coli-Thermus* spp. vector, Km | (Cava et al., 2007) |
| pWUR112/77-1 | Cloning, expression shuttle *E. coli-Thermus* spp. vector, Bl | (Brouns et al., 2005) |
| pKT1 | pUC19 derivative with the *kat* gene cassette encoding thermostable resistance to Km | (Lasa et al., 1992) |
| pET28drpA | Production His-DrpA in *E.coli* | This work |
| pET28drpb | Production His-DrpB in *E.coli* | This work |
| pDOWN1 | pUC derivative with the 3’ region of the *nar* operon and the *drpAB* cluster | (Ramírez-Arcos et al., 1998) |
| pMKE1bgaA | Promoter probe vector with a thermostable beta-galactosidase under the control of *Pnar* | (Moreno et al., 2003) |
| pWURdrpA | Vector for expression of DrpA in *T. thermophilus*, Bl | This work |
| pWURdrpB | Vector for expression of DrpB in *T. thermophilus*, Bl | This work |
| pWURdrpAB | Vector for expression of DrpA and DrpB in *T. thermophilus*, Bl | This work |
| pDrpAkat | Vector for isolation of *drpA::kat* mutants | This work |
| pDrpBkat | Vector for isolation of *drpB::kat* mutants | This work |
| pDrpABkat | Vector for isolation of *drpAB::kat* double mutants | This work |
| pMHnarbgaA | Vector to assay the expression of the *Pnar* promoter | (Cava el at 2007) |
| pMHdnrbgaA | Vector to assay the expression of the *Pdnr* promoter | (Cava el at 2007) |
| pMHdrpbgaA | Vector to assay the expression of the *Pdrp* promoter | This work |

**Plasmid construction**

Plasmid pMHdrpbgaA was constructed in two steps. First the region containing the *Pdrp* promoter was amplified from plasmid pDOWN1 strain with primers PdrpNde1 and PdrpXba1 and cloned between the Nde1 and Xba1 sites of the bifucntional *E. coli-Thermus* expression vector pMKEbgaA, which confers thermostable resistance to Kanamycin (Moreno et al 2003). In the second step, the DNA fragment containing the promoter and the *bgaA* gene from this plasmid was isolated by Xba1-HindIII restriction cuts and cloned into the same sites of bifunctional plasmid pMH184, conferring thermostable resistance to Hygromycin.

pWURdrpA and pWURdrpB and pWURdrpAB were also constructed in two steps. First, the drpA, drpB or a DNA fragment containing both genes were amplified with primer pairs NdeIdrpAM3/ revDrpAEcoR1, NdeIdrpB/ revDrpBEcoR1 and NdeIdrpAM3/ revDrpBEcoR1, respectively, and cloned between the NdeI and EcoR1 sites of pET22b. Then, the fragment between Xba1 and Eco R1 of the plasmids obtained was isolated and cloned into the equivalent sites of the bifunctional vector pWUR112/77-1 (Brouns et al., 2005), which confers thermostable resistance to Bleomycin B.

**References to Table S1**

Brouns, S.J., Wu, H., Akerboom, J., Turnbull, A.P., de Vos, W.M., and van der Oost, J. (2005) Engineering a selectable marker for hyperthermophiles. *J Biol Chem* **280**: 11422-11431.

Cava, F., Laptenko, O., Borukhov, S., Chahlafi, Z., Blas-Galindo, E., Gomez-Puertas, P., and Berenguer, J. (2007) Control of the respiratory metabolism of *Thermus thermophilus* by the nitrate respiration conjugative element NCE. *Mol Microbiol* **64**: 630-646.

Lasa, I., Caston, J.R., Fernandez-Herrero, L.A., de Pedro, M.A., and Berenguer, J. (1992) Insertional mutagenesis in the extreme thermophilic eubacteria *Thermus thermophilus* HB8. *Mol Microbiol* **6**: 1555-1564.

Moreno, R., Zafra, O., Cava, F., and Berenguer, J. (2003) Development of a gene expression vector for Thermus thermophilus based on the promoter of the respiratory nitrate reductase. *Plasmid* **49**: 2-8.

Ramírez-Arcos, S., Fernandez-Herrero, L.A., and Berenguer, J. (1998) A thermophilic nitrate reductase is responsible for the strain specific anaerobic growth of *Thermus thermophilus* HB8. *Biochim Biophys Acta* **1396**: 215-227.

Vieira, J., and Messing, J. (1982) The pUC plasmids, an M13mp7-derived system for insertion mutagenesis and sequencing with synthetic universal primers. *Gene* **19**: 259-268.

**Table S2.** Oligonucleotides used in this work

| **NAME** | **SEQUENCE (5>3’)** | **USE** |
| --- | --- | --- |
| PdrpNde1 | AAAACATATGCCATGACCTGGGTCAAGG | Amplification *Pdrp* |
| PdrpXba1 | GCTCTAGACGCCAGGCGGGGGGAGTT | Amplification *Pdrp* |
| NdeIdrpAM3 | AAAACATATGGAGCTGGACGT | Amplification *drpA* |
| revDrpAEcoR1 | CGGAATTCGGACTACTTCTTCCCGAT | Amplification *drpA* |
| NdeIdrpB | GACATATGGCGGGTGCGGA | Amplification *drpB* |
| revDrpBEcoR1 | CGGAATTCCTACCCCCCTTTCCGGAT | Amplification *drpB* |
